# Implementation and preliminary evaluation of an entrepreneurship, biomedical innovation, and design pathway in a school of medicine curriculum

**DOI:** 10.1017/cts.2025.10112

**Published:** 2025-07-24

**Authors:** Nathaniel Hafer, Christian Keenan, Anindita Deb

**Affiliations:** 1 UMass Center for Clinical and Translational Science, UMass Chan Medical School, Worcester, MA, US; 2 Program in Molecular Medicine, UMass Chan Medical School, Worcester, MA, US; 3 TH Chan School of Medicine, UMass Chan Medical School, Worcester, MA, US; 4 Department of Neurology, UMass Chan Medical School, Worcester, MA, US

**Keywords:** I-Corps, Commercialization, engineering, entrepreneurship, medical education

## Abstract

New educational curricula are emerging to train physicians for healthcare in the 21^st^ century. The University of Massachusetts Chan Medical School T.H. Chan School of Medicine (UMass Chan) implemented an MD curriculum redesign in the fall of 2022 that included seven educational pathways, including Entrepreneurship, Biomedical Innovation, and Design. This new pathway curriculum introduces students to the principles of innovation, entrepreneurship, basic engineering principles, and technology commercialization. It is modeled after the I-Corps curriculum with added material regarding engineering principles. I-Corps was initially developed by the National Science Foundation (NSF) to help scientists understand the commercial potential of their inventions. Major elements include the Business Model Canvas and Customer Discovery [19-22]. First-year (Class of 2027) and second-year (Class of 2026) pathway students were invited to participate in online surveys evaluating course material and their knowledge of course content. Initial results show that the program was well received and student self-assessment demonstrated significant improvement. Objective student knowledge also significantly improved. Novel curricula have the potential to transform medical education and prepare future physicians to practice healthcare in the 21^st^ Century.

## Introduction

Healthcare continues to undergo rapid change as technology and societal forces alter the way that we deliver and receive care. In response, new educational approaches have emerged to train the medical workforce. These approaches include active learning, including the use of technology for simulation, and greater emphasis on mobilizing students to learn and respond to community health needs [1]. One type of program that is becoming increasingly popular is focused on innovation and entrepreneurship. Core curricula typically focus on a combination of scientific, business, regulatory, finance, and design topics that teach students how to successfully transfer knowledge into products that benefit society. A growing literature describes the characteristics and goals of these programs in the US, Europe, Japan, and China [2–6].

In August of 2022, the University of Massachusetts Chan Medical School T.H. Chan School of Medicine (UMass Chan) implemented a major curriculum change entitled the “Vista Curriculum.” The goal was to develop a contemporary and innovative curriculum that promotes curiosity and inquiry, empowers learners, and enables future physician leaders to equitably and expertly care for diverse patient populations. All students in this new MD curriculum participate in a “pathway” that provides foundational training in team-based project work and critical thinking combined with pathway-specific knowledge. This pathway-specific training serves as the foundation of a faculty-mentored longitudinal project that spans medical school [7]. Through interactive class sessions and outside research, students complete a group Pathways Longitudinal Project (PLP) throughout their four years of medical school. The PLP is a longitudinal team-driven project with required participation by all students in Vista pathways. These projects may be new initiatives or may build upon existing initiatives that have demonstrated value to UMass Chan. Through the PLP, students strive to make a longitudinal and sustainable impact on patients, the health system, community, or global population. The seven pathways include (1) Structural Inequity, Advocacy, and Justice; (2) Entrepreneurship, Biomedical Innovation, and Design; (3) Clinical Care; (4) Clinical, Community, and Translational Research; (5) Education; (6) Population, Community, and Global Health; and (7) Health Systems Science.

Prior to 2022, the UMass Chan curriculum lacked any formalized curriculum surrounding design thinking, product commercialization, entrepreneurship, or engineering analysis. Many peer institutions have developed design thinking and innovation curricula similar to the UMass Chan pathways model. These peer institutions have various differences, but all include a focus on innovation and new product development. Other topics that are included in many of the peer institution’s options, but are not common to all of them, include: leadership, entrepreneurship, health systems science, and medical technology engineering [8–18]. Given that UMass Chan has its own unique resources, curricular needs, and required competencies for its students, creation of a unique pathway was necessary to properly meet all the needs of this institution.

The process for creating this Entrepreneurship, Biomedical Design, and Innovation pathway included adapting material from the National Science Foundation’s Innovation Corps (I-Corps) program as well as additional lectures about biomedical design, human factors, and hazard analysis, which were identified as important concepts that are not part of the I-Corps curriculum [19–22]. While the I-Corps material covered entrepreneurship and business development aspects thoroughly, there was still a gap in aspects of engineering analysis.

Engineering analysis utilizes multiple sequential steps in the design and prototyping of a solution to any problem and is commonly taught in engineering school curricula and some business school curricula. The focus of this project was to augment the I-Corps curriculum with facets of engineering analysis by way of three additional lectures to the pathways students. The objectives related to engineering analysis included: (1) hazard analysis; (2) engineering design with a focus on inputs, constraints, and systems-level concepts; (3) human factors and how it is used in all steps of designing a solution; (4) assessment of students’ understanding of the content. Engineering content was originally developed by Dr Keenan and has been updated by School of Engineering faculty at UMass Amherst and UMass Lowell.

This manuscript describes the Entrepreneurship, Biomedical Innovation and Design pathway curriculum and provides preliminary evaluation data and learning outcomes. The lessons learned from these data are informing further adjustments to the content and learning objectives of this pathway.

## Materials and methods

Participants described in this manuscript are members of the UMass Chan School of Medicine Class of 2026 (11 students) and 2027 (21 students) in the Entrepreneurship, Biomedical Design, and Innovation pathway. The UMass Chan Institutional Review Board (IRB) determined that the survey questionnaires used in this manuscript are not human subjects research as defined by United States Department of Health and Human Services (DHHS) and Food and Drug Administration (FDA) regulations, therefore a Clinical Trial Number is not applicable (study ID: STUDY00002045). Since this work was determined to be not human subjects research, informed consent to participate in these voluntary surveys was not obtained.

### Pathway and project selection

New medical school students participate in a “Pathways Fair” as part of their orientation. Lead pathway instructors and upper-level students share information about the curriculum and longitudinal projects. Office hours and pathway-specific sessions provide an opportunity to discuss each pathway in greater depth. During the fall semester students submit a ranked list of pathway preferences and are placed into cohorts of roughly the same size, about 20 students/pathway. Pathway leaders work with UMass Chan faculty to identify eligible projects. Students are also allowed, with permission, to develop their own projects. Students self-select into groups of 3–5 and pick projects based on mutual student-faculty interest. Projects range across disease indications and include medical devices, biologics, digital health, and educational programs.

### Course evaluation

Students were sent a link inviting them to complete an electronic survey at the end of the fall and spring semesters hosted on the Online Access to Student Information and Scheduling (OASIS) platform. Students responded to questions on a 4-part Likert scale where 1 = poor, 2 = fair, 3 = good, 4 = excellent. Survey responses were anonymous. The UMass Chan Institutional Research, Evaluation, and Assessment group in the Office of Educational Affairs compiled and analyzed data, then provided reports to instructors.

### Engineering content evaluation

Prior to the engineering course section, all second-year students in the pathway received a pre-assessment survey that included questions about their knowledge of course material. These questions established the students’ baseline knowledge before the lectures were given. The survey was developed (CK), approved by the pathway director (NH), and delivered prior to the first session.

All students received the same three lectures. One focused on hazard analysis, one focused on engineering design, and one focused on human factors analysis. Each lecture included a portion of the allotted time to using the skills just presented and applying them to each students’ longitudinal project.

After the third lecture concluded, the students received a post-assessment survey, comprised of the same questions as the pre-assessment. The post-assessment survey also included questions to assess the students’ perceptions on how the material was presented, how much material was presented, and any opportunities for improvement in the delivery of the curriculum.

The self-assessment questions asked students to rate their level of comfort and their perception of their understanding of the material on a 5-point Likert scale, where 1 was the lowest level of confidence/competence and 5 was the highest. The material assessment comprised of multiple-choice questions; some had four options while others had five. Simple means were calculated for each question of the self-assessment, and as an aggregate. A two-sample *t*-test was performed using those means to assess statistical significance. For the material-based multiple-choice questions, the answers were labeled as either completely correct or incorrect. A distribution of correct and incorrect answers was generated between the pre- and post-survey results, and a chi-squared analysis was used to determine whether the two correct/incorrect distributions were significantly different.

## Results

### Entrepreneurship, biomedical design, and innovation pathway description

This pathway gives students a hands-on customer discovery learning experience that teaches students how to successfully transfer knowledge into products and processes that benefit society. The commercialization and innovation content is adapted from the National Science Foundation’s I-Corps program, the premiere federally-funded innovation and commercialization training in the U.S. Students learn the Business Model Canvas, with a focus on Customer Segments and Value Proposition. Additional content focuses on the patient’s journey through the healthcare system, the U.S. healthcare ecosystem, and insurance, reimbursement, and payment models. Students also learn the customer discovery (interview) process to get the perspective of possible customers, partners, and competitors. This guides students in how to deal with the chaos and uncertainty of commercializing innovations and creating ventures. Interviews lead to real-world insights, assessing key components of the business model, and often lead to pivots or refinements. The course goals are to: (1) provide aspiring physician-entrepreneurs an experiential learning opportunity to help determine the commercial readiness of a technology; (2) connect students to the tools and resources needed to successfully commercialize technology; and (3) develop an understanding of the commercialization process, increasing the ability to lead and play an active role in advancing a technology. The curriculum for the first and second years of this pathway is found in Table 1.

**Table 1.** Curriculum for entrepreneurship, biomedical design, and innovation pathway

Week	Content	Description
**Year 1**		
1	I-Corps approach and Business Model Canvas (BMC) overview Identifying customer segments and value propositions	Introduction Who is the customer for your product? What value does your product deliver to customers?
2	Customer Discovery	How to conduct unbiased interviews with customers
3	Customer Discovery simulation: interviewing patients and providers	Practice interviews in UMass simulation center
4	Workflows and ecosystems in healthcare	Patient journey through healthcare system; competition; large-scale factors in healthcare
5	Critical actions for life science companies	Financial, physical, personnel, and intellectual property resources; partnerships
6	Regulatory vs economic adoption	Regulatory models; market share
7	Minimal viable product and intro to US healthcare reimbursement	Prototypes and design phases; reimbursement
**Year 2**		
1	Distribution channels	How do you get your product to your customer?
2	Customer relationships	How do you get, keep, and grow customers?
3	Intro to Biomedical Design	The intersection of medicine and engineering
4	Intro to hazard analysis	Developing safe products
5	Intro to human factors analysis	Developing products to be used efficiently and as intended
6	Technology transfer and intellectual property	Types of intellectual property and tech transfer strategies

Summary of content presented over the first two years of the Entrepreneurship, Biomedical Design, and Innovation pathway. Each class is 2–3 hours and consists of student presentations about what they’ve learned from customer discovery and instructor presentations about the Business Model Canvas and product development.

The course is designed to promote active learning. Each class includes an assignment to watch videos outside of class time that introduce elements of the Business Model Canvas and Customer Discovery. In-class time is dedicated to student presentations focused on lessons learned from customer interviews and didactic presentations that emphasize key parts of the class content. One session in the first year includes simulated customer interviews in the UMass Chan Simulation Center, where students practice their interview skills with standardized professionals playing the role of a clinician or patient.

### Curriculum evaluation

At the end of each semester students were invited to complete a pathway evaluation survey. Initial results show that the program was well received. 15/20 (75%) first-year students in the Fall 2023 and 13/21 (62%) in the Spring 2024 rated the experience as good or excellent. 8/10 (80%) second-year students rated the experience as good or excellent in both Spring 2023 and Fall 2023. Students were also asked if the curriculum met their expectations. 16/20 (80%) first year students in the Fall 2023 and 13/21 (62%) in the Spring 2024 rated the experience as good or excellent. 8/10 (80%) second-year students rated the experience as good or excellent in both Spring 2023 and Fall 2023. Full results are presented in Table 2.

**Table 2.** Student evaluation of the entrepreneurship, biomedical design, and innovation pathway curriculum

Question	1 = poor *n* (%)	2 = fair *n* (%)	3 = good *n* (%)	4 = excellent *n* (%)
**Class of 2027 students, Fall 2023 (*n* = 20)**				
Overall, how would you rate this pathways experience?	2 (10)	3 (15)	10 (50)	5 (25)
The pathways curriculum met my expectations	2 (10)	2 (10)	13 (65)	3 (15)
**Class of 2027 students, Spring 2024 (*n* = 21)**				
Overall, how would you rate this pathways experience?	2 (10)	6 (29)	6 (29)	7 (33)
The pathways curriculum met my expectations	2 (10)	6 (29)	7 (33)	6 (29)
**Class of 2026 students, Fall 2022 (*n* = 42)**				
Overall, how would you rate the quality of this pathways educator’s curriculum delivery?	1 (2)	7 (17)	24 (57)	10 (24)
Instructor was effective in guiding my interests towards specific pathways content areas	1 (2)	6 (14)	28 (67)	7 (17)
**Class of 2026 students, Spring 2023 (*n* = 11)**				
Overall, how would you rate this pathways experience?	1 (9)	2 (18)	4 (36)	4 (36)
The pathways curriculum met my expectations	0 (0)	3 (27)	4 (36)	4 (36)
**Class of 2026 students, Fall 2023 (*n* = 10)**				
Overall, how would you rate this pathways experience?	0 (0)	2 (20)	2 (20)	6 (60)
The pathways curriculum met my expectations	0 (0)	2 (20)	3 (30)	5 (50)

At the end of the year students were invited to participate in a course survey where they were asked to rate questions according to a 4-point Likert scale where 1 = poor and 4 = excellent. Number of student responses = *n*, % of overall total in parentheses.

### Delivery and preliminary evaluation of engineering course content

Three lectures were provided during the Fall 2023 semester to 11 second-year medical students in the Entrepreneurship, Biomedical Innovation, and Design pathway. Prior to the start of the lecture series, the pre-survey was distributed to all students in this cohort, of which nine filled out the survey. The three unique lectures were delivered weeks apart from one another, where two were delivered over Zoom and one was delivered in-person. The first lecture was entitled “Introduction to Biomedical Design” and was delivered over Zoom, the second lecture was entitled, “Hazard Analysis and Risk Management” and was delivered in-person, and the third lecture was entitled, “Human Factors” and was delivered over Zoom. After the three lectures were delivered, the post-survey was provided to the same 11-student cohort, of which nine students filled it out. A blank copy of the pre-survey is in Appendix 1 and a blank copy of the post-survey is in Appendix 2. The survey responses of the pre-survey are in Appendix 3 and the survey responses of the post-survey are in Appendix 4. The results of the questions where students reflected on their own sense of comfort and understanding are summarized in Table 3. The aggregate average of the students’ responses to each question was compiled along with a standard deviation. A two-sample *t*-test was performed to evaluate whether the differences between the pre-survey results and post-survey results for each question were statistically significant.

**Table 3.** Results of self-assessment of comfort and understanding of engineering curriculum

#	Question	Pre-Test Average ± SD (*n* = 9)	Post-Test Average ± SD (*n* = 9)	*t*-test *p*-value
1	How well do you believe you understand engineering design principles?	2.22 ± 1.39	4.00 ± 0.50	0.001199*
2	How comfortable are you with engineering hazard analysis and associated risk management?	1.33 ± 0.71	4.00 ± 0.50	<0.0001*
3	How comfortable are you with the engineering design process and generating inputs and outputs?	1.67 ± 1.00	3.89 ± 0.60	0.000016*
4	How comfortable are you with creating and using a decision-making matrix to help weigh options in a quantified manner?	1.67 ± 1.12	3.89 ± 0.60	0.000039*
5	How comfortable are you with utilizing human factors analysis in designing new technology?	1.56 ± 0.73	4.22 ± 0.67	<0.00001*

This table presents the average rating that students evaluated themselves before and after the lecture series. Students were presented the same prompts both times and were able to rate their understanding/comfort on a scale from 1 to 5, where 1 was little to no comfort or understanding of the material and 5 was very comfortable or high understanding of the material. Each question was evaluated for statistical differences using a two-sample *t*-test, with the associated p-values in the last column. The * is indicative of statistical significance as the p-value is less than α = 0.05.

In the pre-survey and post-survey, the students were also asked questions about the content of the lectures, which were presented as multiple-choice questions. This was to assess their baseline knowledge and any changes that occurred after the lectures. The performance of the students on each question are presented in Table 4. The results were then analyzed by a Chi-Squared test to see if there was a statistically significant difference in the distribution of correct and incorrect answers before and after the lecture series. The associated p-value of <0.0001 is less than the *α* = 0.05, indicating a statistically significant difference in score distributions.

**Table 4.** Changes in student knowledge before and after delivery of the engineering course content

#	Question	Correct Answer	Correct or Incorrect?	Pre-Test Count	Post-Test Count
1	The process of determining if design outputs reach the design input goals is called:	Verification	Correct	5	9
			Incorrect	4	0
2	The process of testing whether a device meets a users' needs and wants, even if it reaches all of the design input goals, is called:	Validation	Correct	6	7
			Incorrect	3	2
3	What are the three components to a problem statement	The problem, the affected population, and the harm	Correct	2	7
			Incorrect	7	2
4	Besides the hazard and harm, what is the third component for initial hazard analysis	The situation of use	Correct	2	8
			Incorrect	7	1
5	When assessing the impact of a hazard, what are the two most important factors for consideration?	Severity of damage and likelihood of damage	Correct	4	7
			Incorrect	5	2
		df	9	χ2	50.964
				*p*-value	<0.0001

The table summarizes the number of correct and incorrect answers for each of the five knowledge assessment questions, before and after the lecture series occurred. The results were evaluated for a statistically significant difference using a Chi-Squared analysis, where the pre-test results were treated as the expected baseline. The associated *p*-value of <0.0001 is less than the *α* = 0.05, indicating a statistically significant difference in score distributions.

The post-survey also included questions regarding the delivery and content of the lecture series. Students were asked to rate how much they agreed with various prompts and provide a rating of whether the amount of material delivered in each lecture was too much, too little, or just right. All respondents either agreed/strongly agreed with the statements, “the lectures provided were clear and easy to follow,” “the lectures provided were helpful and can be applied to my project,” and “the lectures provided were helpful and can be applied to my career as a physician.” All 9 respondents felt that the lectures presented provided students with the right depth of understanding of the material.

Lastly, the post-survey also provided optional free-text opportunities for students to provide their narrative feedback on aspects that they enjoyed, and aspects for improvement that they would like to see. Of the nine responses, three provided responses to these optional questions. While a few of the comments provided praise of the lectures and the corresponding lecturer, there were a few notable quotes. One student remarked, “These were incredibly helpful lectures for our projects especially for students, like myself, with no engineering experience.” Another remarked, “I loved the stories you added in to make it funny and engaging. I wish there were more diagrams.”

## Discussion

Medical education is evolving in response to rapid changes in the healthcare system. This manuscript describes a new pathway curriculum created to introduce students to the principles of innovation, entrepreneurship, and technology commercialization. While it is still too early to measure long-term outcomes of student learning and career choices, initial feedback and evaluations suggest that student learning and acceptance of the program is high.

The following lessons learned may facilitate the implementation of similar programs. We found it helpful to work with our technology transfer office to identify projects that could benefit from a commercialization analysis. Tech transfer professionals are also a good source of instructors and project mentors. It is also important to establish close communication with faculty mentors to clarify expectations and time commitments. Some faculty assume they are mentoring wet lab research projects rather than studies of the commercialization potential of lab research. We also recommend students work in groups rather than on individual projects. A team approach helps busy students move projects forward, especially once students enter their clinical rotations. Finally, program instructors should expect student teams to not make progress and conduct customer discovery interviews as rapidly as traditional I-Corps teams. Unlike traditional teams that work full-time on their startup venture, many students have not been previously introduced to these concepts and are juggling a pathway project with course work and clinical responsibilities.

The engineering lecture series had the goal of increasing engineering education in the medical school curriculum and delivering the content in a way that was engaging and led to educational benefits. Evaluation and student comments to date indicate that students enjoy the curriculum and appreciated its delivery. The results in Table 3 show that there was a noticeable increase in the students’ levels of comfort and self-assessed understandings of the material, and the results in Table 4 show that students’ knowledge of the material significantly improved after the three lectures. One limitation of these data are the small sample sizes, which are discussed in greater detail below. Student feedback from the lecture series suggests that the content was applicable to their projects and future career plans.

As mentioned above, the small sample size of 9 students completing the engineering content is a limitation. As additional student classes complete the program we will accumulate short- and long-term data related to learning metrics and expand our statistical analyses. We are encouraged by initial results showing increases in student knowledge and comfort in understanding the engineering curriculum. The growth of the program from 11 students in the Class of 2026 to 21 students in the Class of 2027 is also a positive trend.

This new pathway curriculum at UMass Chan introduces students to the principles of innovation, entrepreneurship, and technology commercialization. An element of this pathway focused on basic engineering principles provided students with baseline understandings of biomedical design, human factors, and risk/hazard analysis. Despite small sample sizes impacting the power and generalizability of the results, the results show improvements in student comfort with the material, students’ knowledge of the material, and their enjoyment and applicability of the curriculum. As additional cohorts move through the curriculum, our evaluation will continue to measure student satisfaction and learning outcomes. Another consideration for future study is the student population. This manuscript is solely focused on the students in the Entrepreneurship, Biomedical Design, and Innovation pathway. Future work will evaluate student perceptions and learning related to the other six pathways offered by UMass Chan. A final consideration is to expand the engineering content. Seeing as engineering analysis is quite vast, three lectures certainly does not cover all the applicable content that students may need. Material that could be introduced includes prototyping, experimental approaches to test prototype quality, and the FDA regulation of medical devices.

## Supporting information

10.1017/cts.2025.10112.sm001 Hafer et al. supplementary material Hafer et al. supplementary material

## Data Availability

All data generated and analyzed during this study are included in this published article and its appendix information files.

## Supplementary material

The supplementary material for this article can be found at https://doi.org/10.1017/cts.2025.10112.
