# Supplementary material for: Implementation and preliminary evaluation of an entrepreneurship, biomedical innovation, and design pathway in a school of medicine curriculum
Source: J Clin Transl Sci. 2025 Jul 24;9(1):e172. doi: 10.1017/cts.2025.10112 (PMC12444688; doi:10.1017/cts.2025.10112)
Supplement: Hafer et al. supplementary material [file S205986612510112Xsup001.docx]

**Appendix**

**Appendix 1. Blank Pre-Survey**

**Engineering Design Curriculum Pre-Survey**

This is part of a Capstone project by Christian Keenan. Your involvement in this survey is completely voluntary and anonymous, but is also greatly appreciated. This pre-survey is to establish a baseline of understanding of biomedical engineering design principles, with the hopes that students' understandings and knowledge of the subject increase over the 3 lectures.

*Page 1:Consenting to participation*

**Q) Please indicate if you agree to take part in this pre and post lecture series survey.**

- I agree
- I disagree

*Page 2: Your Identifier*

Your Unique Identifier

In order for students to submit this form in an anonymous way, while allowing us to analyze individual students' growth and knowledge expansion, we are creating unique identifiers. Please use these rules for the typing your’s in:

- Your two-digit date of birth (ex. "08" if you were born on March 8, "23" if you were born on June 23)

- The first two digits of your address (ex. "01" if you live at 1 Main St, "25" if you live at 25 Main St, "31" if you live at 3128 Main St)

- The last two digits of your cell phone number (ex. "98" if your number is 555-555-5598)

- Your age as of October 1, 2023 (if it is your birthday, please put the age that you just turned)

**Q) Please include your unique identifier.**

*(free text answer box available)*

*Page 3: Assessing Students’ Comfort*

This section will focus on determining each students’ level of comfort with the material

**Q1) How well do you believe you understand engineering design principles?**

- 1: Not at all
- 2
- 3
- 4
- 5: Very well

**Q2) How comfortable are you with engineering hazard analysis and associated risk management?**

- 1: Not at all
- 2
- 3
- 4
- 5: Very comfortable

**Q3) How comfortable are you with the engineering design process and generating inputs and outputs?**

- 1: Not at all
- 2
- 3
- 4
- 5: Very comfortable

**Q4) How comfortable are you with creating and using a decision-making matrix to help weigh options in a quantified manner?**

- 1: Not at all
- 2
- 3
- 4
- 5: Very comfortable

**Q5) How comfortable are you with utilizing human factors analysis in designing new technology?**

- 1: Not at all
- 2
- 3
- 4
- 5: Very comfortable

*Page 4: Assessing Students’ Knowledge*

These are questions to assess baseline knowledge in the area. Please do not use any outside resources when answering them. These are topics that will be covered in the lecture series.

**Q1) The process of determining if design outputs reach the design input goals is called:** *(select 1)*

- Ascertainment
- Experimentation
- Error analysis
- Verification
- Prototyping

**Q2) The process of testing whether a device meets a users’ needs and wants, even if it reaches all of the design input goals, is called:** *(select 1)*

- Prototyping
- Validation
- Corrective Design
- Authentication
- Due Diligence

**Q3) What are the three components to a problem statement?** *(select 1)*

- The problem, your designs, and the cost
- The problem, the user, and the prototypes
- The problem, the affected population, and the harm
- The problem, the current solutions, and the flaws of the current solutions

**Q4) Besides the hazard and harm, what is the third component for initial hazard analysis and identification?** *(select 1)*

- The location
- The user
- Others involved that aren’t the user
- The situation

**Q5) When assessing the impact of a hazard, what are the two most important factors for consideration?** *(select 2)*

- Cost
- Location
- People involved
- Likelihood
- Severity of damage
- Stakeholders

*Page 5: Remaining Questions*

**Q) Do you have any remaining questions or comments?**

*(Free text box available)*

**Appendix 2. Blank Post-Survey**

**Engineering Design Curriculum Post-Survey**

This is part of a Capstone project by Christian Keenan. Your involvement in this survey is completely voluntary and anonymous, but is also greatly appreciated. This post-survey is meant to track how understanding and comfort with the material changed with the addition of the 3 lectures. It is almost completely identical to the pre-survey, with the addition of some feedback questions at the end.

*Page 1:Consenting to participation*

**Q) Please indicate if you agree to take part in this pre and post lecture series survey.**

- I agree
- I disagree

*Page 2: Your Identifier*

Your Unique Identifier

In order for students to submit this form in an anonymous way, while allowing us to analyze individual students' growth and knowledge expansion, we are creating unique identifiers. Please use the rules for the typing your's in:

- Your two-digit date of birth (ex. "08" if you were born on March 8, "23" if you were born on June 23)

- The first two digits of your address (ex. "01" if you live at 1 Main St, "25" if you live at 25 Main St, "31" if you live at 3128 Main St)

- The last two digits of your cell phone number (ex. "98" if your number is 555-555-5598)

- Your age as of October 1, 2023 (if it is your birthday, please put the age that you just turned)

**Q) Please include your unique identifier.**

*(free text answer box available)*

*Page 3: Assessing Students’ Comfort*

This section will focus on determining each students’ level of comfort with the material

**Q1) How well do you believe you understand engineering design principles?**

- 1: Not at all
- 2
- 3
- 4
- 5: Very well

**Q2) How comfortable are you with engineering hazard analysis and associated risk management?**

- 1: Not at all
- 2
- 3
- 4
- 5: Very comfortable

**Q3) How comfortable are you with the engineering design process and generating inputs and outputs?**

- 1: Not at all
- 2
- 3
- 4
- 5: Very comfortable

**Q4) How comfortable are you with creating and using a decision-making matrix to help weigh options in a quantified manner?**

- 1: Not at all
- 2
- 3
- 4
- 5: Very comfortable

**Q5) How comfortable are you with utilizing human factors analysis in designing new technology?**

- 1: Not at all
- 2
- 3
- 4
- 5: Very comfortable

*Page 4: Assessing Students’ Knowledge*

These are questions to assess new knowledge in the area. Please do not use any outside resources when answering them. These are topics that were covered in the lecture series.

**Q1) The process of determining if design outputs reach the design input goals is called:** *(select 1)*

- Ascertainment
- Experimentation
- Error analysis
- Verification
- Prototyping

**Q2) The process of testing whether a device meets a users’ needs and wants, even if it reaches all of the design input goals, is called:** *(select 1)*

- Prototyping
- Validation
- Corrective Design
- Authentication
- Due Diligence

**Q3) What are the three components to a problem statement?** *(select 1)*

- The problem, your designs, and the cost
- The problem, the user, and the prototypes
- The problem, the affected population, and the harm
- The problem, the current solutions, and the flaws of the current solutions

**Q4) Besides the hazard and harm, what is the third component for initial hazard analysis and identification?** *(select 1)*

- The location
- The user
- Others involved that aren’t the user
- The situation

**Q5) When assessing the impact of a hazard, what are the two most important factors for consideration?** *(select 2)*

- Cost
- Location
- People involved
- Likelihood
- Severity of damage
- Stakeholders

*Page 5: Feedback on Lectures*

This portion is completely optional, but highly encouraged. Please rate how much you agree/disagree with the statements below.

**Q1) The lectures that Christian provided were clear and easy to follow.** *(Select 1)*

- Strongly disagree
- Disagree
- Neutral
- Agree
- Strongly agree

**Q2) The lectures that Christian provided were helpful and can be applied to my project.** *(Select 1)*

- Strongly disagree
- Disagree
- Neutral
- Agree
- Strongly agree

**Q3) The lectures that Christian provided were helpful and can be applied to my career as a physician.** *(Select 1)*

- Strongly disagree
- Disagree
- Neutral
- Agree
- Strongly agree

**Q4) The lectures that Christian presented provided me with the right depth of understanding of the material.** *(Select 1)*

- Definitely not enough depth of the material
- Slightly not enough depth of the material
- Just right
- A bit too much depth of the material
- Way too much depth of the material

**Q5) Is there anything that you would like changed, improved upon, or enhanced regarding these presentations?**

*(Free text box available)*

**Q6) Do you have any other feedback for Christian and his presentations?**

*(Free text box available)*

*Page 6: Remaining Questions*

**Q) Do you have any remaining questions or comments?**

*(Free text box available)*

**Appendix 3. Pre-Survey Results**

| **Question** | **Entry 1** | **Entry 2** | **Entry 3** | **Entry 4** | **Entry 5** | **Entry 6** | **Entry 7** | **Entry 8** | **Entry 9** |
| --- | --- | --- | --- | --- | --- | --- | --- | --- | --- |
| Please indicate if you agree to take part in this pre and post lecture series survey | I agree | I agree | I agree | I agree | I agree | I agree | I agree | I agree | I agree |
| Please include your unique identifier | 13305023 | 16415626 | 21727323 | 25797626 | 29922623 | 01523426 | 02698823 | 03338523 | 0622622738 |
| How well do you believe you understand engineering design principles? | 1 | 1 | 4 | 4 | 2 | 2 | 1 | 4 | 1 |
| How comfortable are you with engineering hazard analysis and associated risk management? | 1 | 1 | 1 | 2 | 1 | 1 | 1 | 3 | 1 |
| How comfortable are you with the engineering design process and generating inputs and outputs? | 1 | 1 | 3 | 3 | 1 | 1 | 1 | 3 | 1 |
| How comfortable are you with creating and using a decision-making matrix to help weigh options in a quantified manner? | 1 | 1 | 4 | 2 | 1 | 1 | 1 | 3 | 1 |
| How comfortable are you with utilizing human factors analysis in designing new technology? | 1 | 2 | 1 | 2 | 1 | 2 | 1 | 3 | 1 |
| The process of determining if design outputs reach the design input goals is called: | Verification | Verification | Experimentation | Verification | Error Analysis | Verification | Experimentation | Verification | Error Analysis |
| The process of testing whether a device meets a users' needs and wants, even if it reaches all of the design input goals, is called: | Authentication | Validation | Validation | Validation | Corrective Design | Validation | Authentication | Validation | Validation |
| What are the three components to a problem statement | The problem, the current solutions, and the flaws with the current solutions | The problem, the user, and the prototypes | The problem, the current solutions, and the flaws with the current solutions | The problem, the affected population, and the harm | The problem, the user, and the prototypes | The problem, the current solutions, and the flaws with the current solutions | The problem, the user, and the prototypes | The problem, the affected population, and the harm | The problem, the user, and the prototypes |
| Besides the hazard and harm, what is the third component for initial hazard analysis | The situation | The user | The user | The user | The user | The user | The location | The situation | The location |
| When assessing the impact of a hazard, what are the two most important factors for consideration? | Cost, Severity of damage | Cost, People Involved | People Involved, Severity of damage | People Involved, Severity of damage | Likelihood, Severity of damage | Likelihood, Severity of damage | Likelihood, Severity of damage | Likelihood, Severity of damage | Cost, People involved |
| Do you have any remaining questions or comments? |  |  |  |  |  |  |  |  |  |

Correct answers are in blue, and incorrect answers are in red

**Appendix 4. Post-Survey Results**

| **Question** | **Entry 1** | **Entry 2** | **Entry 3** | **Entry 4** | **Entry 5** | **Entry 6** | **Entry 7** | **Entry 8** | **Entry 9** |
| --- | --- | --- | --- | --- | --- | --- | --- | --- | --- |
| Please indicate if you agree to take part in this pre and post lecture series survey | I agree | I agree | I agree | I agree | I agree | I agree | I agree | I agree | I agree |
| Please include your unique identifier | 13305023 | 16415626 | 25797626 | 29922623 | 01523426 | 02698823 | 03338523 | 05235326 | 06010024 |
| How well do you believe you understand engineering design principles? | 4 | 4 | 4 | 4 | 4 | 4 | 4 | 3 | 5 |
| How comfortable are you with engineering hazard analysis and associated risk management? | 4 | 4 | 4 | 4 | 4 | 4 | 4 | 3 | 5 |
| How comfortable are you with the engineering design process and generating inputs and outputs? | 3 | 4 | 4 | 4 | 4 | 4 | 4 | 3 | 5 |
| How comfortable are you with creating and using a decision-making matrix to help weigh options in a quantified manner? | 3 | 4 | 4 | 4 | 4 | 4 | 4 | 3 | 5 |
| How comfortable are you with utilizing human factors analysis in designing new technology? | 4 | 4 | 4 | 4 | 5 | 5 | 4 | 3 | 5 |
| The process of determining if design outputs reach the design input goals is called: | Verification | Verification | Verification | Verification | Verification | Verification | Verification | Verification | Verification |
| The process of testing whether a device meets a users' needs and wants, even if it reaches all of the design input goals, is called: | Validation | Validation | Validation | Validation | Authentication | Validation | Validation | Validation | Prototyping |
| What are the three components to a problem statement | The problem, the affected population, and the harm | The problem, the affected population, and the harm | The problem, the affected population, and the harm | The problem, the affected population, and the harm | The problem, the affected population, and the harm | The problem, the user, and the prototypes | The problem, the affected population, and the harm | The problem, the user, and the prototypes | The problem, the affected population, and the harm |
| Besides the hazard and harm, what is the third component for initial hazard analysis | The situation | The situation | The situation | The situation | The situation | The situation | The situation | The user | The situation |
| When assessing the impact of a hazard, what are the two most important factors for consideration? | Likelihood, Severity of damage | Likelihood, Severity of damage | Likelihood, Severity of damage | Likelihood, Severity of damage | Likelihood, Severity of damage | Likelihood, Severity of damage | Likelihood, Severity of damage | Likelihood, Stakeholders | People involved, Severity of damage |
| The lectures that Christian provided were clear and easy to follow | Strongly agree | Strongly agree | Strongly agree | Strongly agree | Strongly agree | Strongly agree | Agree | Agree | Strongly agree |
| The lectures that Christian provided were helpful and can be applied to my project | Agree | Agree | Strongly agree | Strongly agree | Strongly agree | Strongly agree | Agree | Agree | Strongly agree |
| The lectures that Christian provided were helpful and can be applied to my career as a physician | Strongly agree | Agree | Agree | Strongly agree | Strongly agree | Strongly agree | Agree | Agree | Strongly agree |
| The lectures that Christian presented provided me with the right depth of understanding of the material | Just right | Just right | Just right | Just right | Just right | Just right | Just right | Just right | Just right |
| Is there anything that you would like changed, improved upon, or enhanced regarding these presentations? |  |  |  | no |  | I loved the stories you added in to make it funny and engaging. I wish there were more diagrams |  |  |  |
| Any other feedback for Christian and his presentations? |  |  | Outstanding job! Thank you! | These were incredibly helpful lectures for our projects especially for students, like myself, with no engineering experience. |  | So useful! You are a real expert! |  |  |  |
| Do you have any remaining questions or comments? |  |  |  | no |  |  |  |  |  |

Correct answers are in blue, and incorrect answers are in red
